# A Novel Ensemble Framework for Comprehensive Early-Stage Colorectal Cancer Diagnosis, Prognosis, and Treatment: Integration of Gastroenterology-Specific Transformer Language Models and Multiple Decision Trees

**DOI:** 10.3390/jcm14134467

**Published:** 2025-06-23

**Authors:** Cem Simsek, Mete Ucdal, Suayib Yalcin, Derya Karakoc

**Affiliations:** 1 Basic Surgical Research, Institute of Health Sciences, Hacettepe University, 06230 Ankara, Turkey; 2 Division of Gastroenterology, Faculty of Medicine, Hacettepe University, 06230 Ankara, Turkey; 3 Division of Internal Medicine, Faculty of Medicine, Hacettepe University, 06230 Ankara, Turkey; 4 Division of Medical Oncology, Faculty of Medicine, Hacettepe University, 06230 Ankara, Turkey; 5 Department of General Surgery, Faculty of Medicine, Hacettepe University, 06230 Ankara, Turkey

**Keywords:** colorectal cancer, artificial intelligence, prognosis prediction

## Abstract

**Background:** Colorectal cancer (CRC) remains a significant global health burden, with early detection and intervention crucial for improving patient outcomes. This study aims to develop and evaluate a novel proof-of-concept ensemble framework combining transformer-based language models and decision tree-based models for early-stage CRC screening, diagnosis, and prognosis. **Methods:** The ensemble framework consists of four key components: (1) GastroGPT, a transformer-based language model for extracting relevant data points from patient histories; (2) a decision tree-based model for assessing CRC risk and recommending colonoscopy; (3) GastroGPT for extracting data points from early CRC patients’ histories; and (4) a suite of decision tree-based models for predicting survival outcomes in early-stage CRC patients. The study employed a retrospective, observational, methodological design using simulated patient cases. **Results:** GastroGPT demonstrated high accuracy in extracting relevant data points from patient histories. The decision tree-based model for CRC risk assessment achieved an area under the receiver operating characteristic curve (AUC-ROC) of 0.85 (95% CI: 0.78–0.92) in predicting the need for colonoscopy. The decision tree-based models for survival prediction showed strong performance, with C-indices ranging from 0.71 to 0.75 for overall survival and disease-free survival at 24, 36, and 48 months. **Conclusions:** The novel ensemble framework demonstrates promising performance in early-stage CRC screening, diagnosis, and prognosis. Further research is needed to validate the models using larger, real-world datasets and to assess their clinical utility in prospective studies.

## 1. Introduction

Colorectal cancer (CRC) remains a significant global health burden, with an estimated 1.9 million new cases and 935,000 deaths in 2020 [1]. Despite advances in screening, diagnosis, and treatment, CRC continues to pose substantial challenges to healthcare systems worldwide, particularly in developed countries where incidence rates are highest [2]. Early detection and intervention are critical for improving patient outcomes, as the 5-year survival rate for localized CRC is approximately 90%, compared to only 14% for metastatic disease [3].

The diagnostic landscape for colorectal cancer presents significant clinical challenges characterized by delayed detection and evolving epidemiological patterns. The symptomatic presentation of colorectal cancer frequently manifests through non-specific clinical indicators, including abdominal discomfort, involuntary weight reduction, and alterations in defecation patterns. These ambiguous symptoms contribute to substantial diagnostic delays, with temporal intervals extending to 9.6 weeks between initial symptom manifestation and specialist consultation, subsequently prolonging the trajectory toward definitive diagnosis and adversely affecting survival prognosis [4]

The current armamentarium of conventional diagnostic modalities encompasses colonoscopy, fecal occult blood testing (including guaiac-based and immunochemical variants), and computed tomographic colonography, each presenting distinct clinical advantages and inherent limitations. Colonoscopy maintains its position as the diagnostic gold standard, demonstrating superior sensitivity, comprehensive colonic visualization capabilities, and the unique advantage of concurrent therapeutic intervention through polypectomy and tissue sampling. Nevertheless, this modality presents significant drawbacks, including procedural invasiveness, mandatory bowel preparation protocols, inherent risks of intestinal perforation and hemorrhagic complications, and substantial associated healthcare expenditures [5]. Fecal occult blood testing methodologies offer non-invasive screening alternatives with favorable cost-effectiveness profiles and administrative simplicity. However, these approaches exhibit diminished sensitivity for adenomatous lesions, generate false-positive results necessitating confirmatory colonoscopy, and require adherence to regular screening intervals to maintain diagnostic efficacy [6]. Computed tomographic colonography represents an intermediate approach, providing less invasive visualization without sedation requirements, though it necessitates bowel preparation, involves radiation exposure, and positive findings mandate subsequent conventional colonoscopy for definitive therapeutic intervention [7]. The inherent trade-offs among diagnostic accuracy, procedural safety, economic considerations, and patient acceptability within existing modalities underscore the critical requirement for innovative diagnostic paradigms. The development of non-invasive, high-precision diagnostic technologies and protocols that demonstrate scalability across heterogeneous healthcare infrastructures represents a paramount objective in advancing colorectal cancer detection and management strategies [8].

Current challenges in CRC management include the non-specific nature of early symptoms, which often overlap with common conditions such as irritable bowel syndrome [9]. This can lead to delayed diagnosis and unnecessary colonoscopies, straining healthcare resources and exposing patients to potential risks [10]. Accurately identifying patients with early-stage CRC is crucial for timely intervention and improved outcomes. However, the heterogeneity of patient populations and the complexity of risk factors make personalized risk assessment and treatment planning challenging [11].

In the era of precision medicine, novel biomarkers and diagnostic tools, such as liquid biopsies, are emerging as promising approaches to improve the accuracy and efficiency of CRC screening and diagnosis [12]. Additionally, a growing number of targeted therapies and immunotherapies are becoming available for CRC treatment, offering the potential for personalized treatment strategies [13]. However, the increasing volume and complexity of patient data, coupled with the scarcity and high cost of these novel therapeutics, present new challenges for clinical decision-making and resource allocation [14].

Artificial intelligence (AI) has the potential to address these challenges by leveraging large-scale patient data to develop predictive models and decision support tools for CRC management [15]. AI-based approaches can integrate multiple data types, including clinical, genomic, and imaging data, to provide comprehensive and personalized risk assessments and treatment recommendations [16]. Moreover, AI-assisted decision support systems can help democratize and standardize care by providing evidence-based guidance to healthcare providers, particularly in resource-limited settings [17].

The present study aims to develop and evaluate a novel proof-of-concept ensemble framework combining transformer-based language models and decision tree-based models for early-stage CRC screening, diagnosis, and prognosis. The framework consists of four key components: (1) GastroGPT, a transformer-based language model for extracting relevant data points from symptomatic patients’ histories; (2) XGBoost, a decision tree-based model for assessing CRC risk and recommending colonoscopy; (3) GastroGPT for extracting data points from early CRC patients’ histories; and (4) a suite of decision tree-based models (XGBoost, LGBoost, Deep Surv, and DeepHit) for predicting survival outcomes in early-stage CRC patients.

## 2. Methods

### 2.1. Study Design and Population

**Figure 1:**
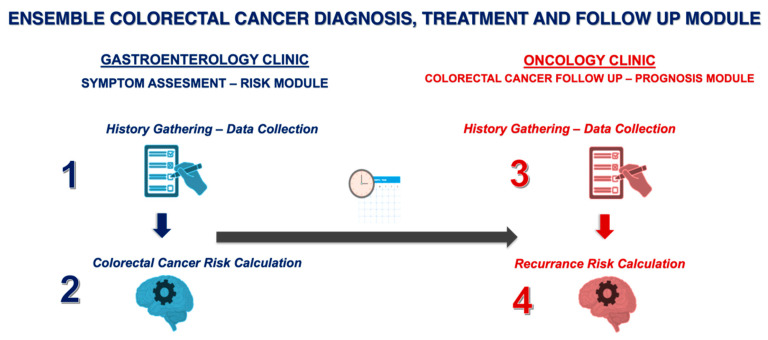
Outline of the Ensemble Colorectal Cancer Diagnosis, Treatment and Follow-up Module. Gastroenterology Clinic—Symptom Assessment and Risk Module a. History Gathering—Data Collection (Step 1) b. Colorectal Cancer Risk Calculation (Step 2). Oncology Clinic—Colorectal Cancer Follow-up and Prognosis Module a. History Gathering—Data Collection (Step 3) b. Recurrence Risk Calculation (Step 4).

This retrospective, observational, methodological study aimed to develop and evaluate a proof-of-concept ensemble decision tree transformer framework for automated screening, diagnosis, and prognosis of early-stage CRC. Inclusion criteria were as follows: (1) patients presenting to the outpatient gastroenterology clinic with non-acute abdominal pain and changes in bowel habits, who underwent colonoscopy, and were aged >18 years; (2) patients diagnosed with stage II or III CRC based on the American Joint Committee on Cancer (AJCC) TNM classification system. Exclusion criteria included insufficient demographic, clinical, or survival data, presence of other malignancies, inflammatory bowel disease, or hereditary CRC syndromes. The sample size was determined based on the widely accepted rule in machine learning, which suggests having at least 10 cases per feature for each model [18]. With 15 features planned for the models (including demographics, clinical parameters, and imaging features), a minimum of 150 cases were required. The ensemble framework consists of four key components, as illustrated in Figure 1. The inclusion criteria for this study were defined as follows: (i) patients aged 18 years or older, (ii) patients undergoing colonoscopy for evaluation of acute or chronic lower gastrointestinal symptoms, and (iii) patients with histo-pathologically confirmed stage II or III colorectal cancer according to the AJCC TNM classification system.

The exclusion criteria comprised the following: (i) patients with missing demographic, clinical, or survival data, (ii) patients with a history of other malignancies, (iii) patients with known inflammatory bowel disease, and (iv) patients with a family history of hereditary colorectal cancer syndromes.

Stage I cases were not included in our study because surgical resection is the only treatment modality that provides curative success in early-stage localized CRC; stage IV cases were excluded considering the presence of metastatic disease and the need for palliative systemic therapy. In this context, our model primarily focuses on stage II and III patients, where decisions about adjuvant chemotherapy after surgery are critical. Inclusion of stage I and IV cases in the model would require separate clinical decision processes and integration of biomarker inputs that would reflect the selection criteria for surgery alone and palliative systemic therapy

This research was approved by the Hacettepe University Non-Interventional Clinical Research Ethics Committee (Approval Code: GO 21/226, dated 16 December 2021). Additionally, for the GastroGPT framework development, ethics approval was obtained for *Defining the Success of Retrospective Artificial Intelligence Language Models in Gastroenterology, Endoscopy and Hepatology Cases* (Approval Code: SBA 24/717). The study was exempt from informed consent requirements due to its retrospective design and use of anonymized data. The study was conducted in accordance with the principles of the Declaration of Helsinki. All patient data were anonymized and handled in compliance with institutional data protection regulations to ensure participant confidentiality throughout the research process. All case summaries were anonymized and assigned random codes before being provided to the two reviewing gastroenterologists. Reviewers had no access to patient identifiers, study group assignments (e.g., real vs. simulated data), or each other’s assessments. The statistical analysis team also worked from the same coded dataset and remained blinded between to the origin and sequence of cases until all analyses were complete.

### 2.2. Development of the Decision Tree Transformer Ensemble Framework

1. Development of the Simulated Trainer Datasets

To enhance diversity in our training cohorts, we supplemented real-world data with systematically generated synthetic cases. We first designed baseline clinical vignettes reflecting typical early-stage CRC presentations, including alarm symptoms (e.g., weight loss, hematochezia) and key risk factors (e.g., family history). We then introduced parameterized variations—sampling age, BMI, tumor grade, and symptom onset from distributions anchored in both our local dataset and established epidemiological ranges. Each synthetic profile was vetted for clinical plausibility by experts, removing or adjusting cases that were not consistent with recognized disease patterns. This semi-automated permutation process generated distinct but credible patient records, which we cross-referenced with our real dataset to ensure alignment in overall variable distributions (e.g., age ranges, MSI status proportions). Through this method, we created 700 synthetic cases for colonoscopy risk modeling and 500 for early CRC prognostication extending our training corpus. Of note, synthetic cases are only used for model fine tuning for GastroGPT, and for decision trees.

2. Transformer Model for Data Extraction from Symptomatic Patients’ Histories

GastroGPT, a transformer-based natural language processing model, was developed to extract relevant data points from the histories of symptomatic patients presenting to an outpatient gastroenterology clinic. The model architecture was based on the BERT (Bidirectional Encoder Representations from Transformers) model, which has demonstrated state-of-the-art performance in various natural language processing tasks [19]. GastroGPT was pre-trained on a large corpus of medical literature, including textbooks, guidelines, and research articles, to acquire a comprehensive understanding of medical terminology and context.

3. Decision Tree-Based Model for CRC Risk Assessment and Colonoscopy Recommendation

A gradient boosting decision tree model, XGBoost, was employed to assess the risk of CRC based on the data points extracted by GastroGPT from symptomatic patients’ histories. The model was trained using the data points extracted from 700 simulated patient cases in the training dataset. The features used for training included age, gender, BMI, family history of CRC, presence of rectal bleeding, weight loss, change in bowel habits, abdominal pain, anemia, low ferritin and low albumin (Table 1).

### 2.3. GastroGPT for Data Extraction from Early CRC Patients’ Histories

For patients diagnosed with early-stage CRC (stage II or III), GastroGPT was applied to extract relevant data points from their medical histories. The model architecture and pre-training process were similar to that used for symptomatic patients. However, the dataset consisted of actual histories of patients diagnosed with early-stage CRC, annotated by the panel of gastroenterologists to highlight relevant prognostic factors, such as tumor location, histological grade, presence of lympho-vascular invasion, and microsatellite instability status.

4. Decision Tree-Based Models for Prognosis and Treatment Response Prediction

A suite of decision tree-based models, including XGBoost, LGBoost, Deep Surv, and DeepHit, was developed to predict prognosis and treatment response in early-stage CRC patients. These models were trained using the data points extracted by GastroGPT from the 500 simulated early-stage CRC patient cases in the training dataset. The features used for training included age, gender, BMI, tumor location, tumor size, histological grade, depth of invasion, number of lymph nodes involved, presence of lympho-vascular invasion, microsatellite instability status, and any relevant laboratory findings, such as carcinoembryonic antigen (CEA) levels.

### 2.4. Data Collection and Preprocessing

The study utilized data from a cohort of patients diagnosed with stage II and III CRC at our institution between January 2010 and December 2020. The electronic health records of these patients were retrospectively reviewed to extract relevant clinical information. A standardized data collection template was followed to ensure consistency in data structure and granularity across all patient cases.

The initial dataset consisted of 1253 patients with biopsy-proven CRC. After applying the inclusion and exclusion criteria, a total of 130 patient cases were included in the final dataset. The collected data were preprocessed to ensure data quality and consistency, including data cleaning, normalization, and encoding.

### 2.5. Evaluation of the Models’ Performance

The performance of the GastroGPT model in extracting relevant data points from patient histories was evaluated using precision, recall, and F1-score metrics. The extracted data points were compared against the annotations provided by the panel of gastroenterologists, serving as the ground truth. The performance of the XGBoost model for CRC risk assessment and colonoscopy recommendation was evaluated using the area under the receiver operating characteristic curve (AUROC), sensitivity, specificity, positive predictive value (PPV), and negative predictive value (NPV). The performance of the decision tree-based models for prognosis and treatment response prediction was evaluated using the concordance index (C-index), time-dependent area under the receiver operating characteristic curve (TD-AUROC), and calibration plots.

Descriptive statistics were used to summarize the characteristics of the simulated patient cases. The 95% confidence intervals for the performance metrics were calculated using bootstrapping with 1000 replicates. The statistical significance of the differences in performance between the developed models and the current standard of care was assessed using paired t-tests for continuous metrics and McNemar’s test for categorical metrics. Subgroup analyses were performed to evaluate the models’ performance across different case complexities, symptom frequencies, and disease stages.

## 3. Results

### 3.1. Study Population

**Figure 2:**
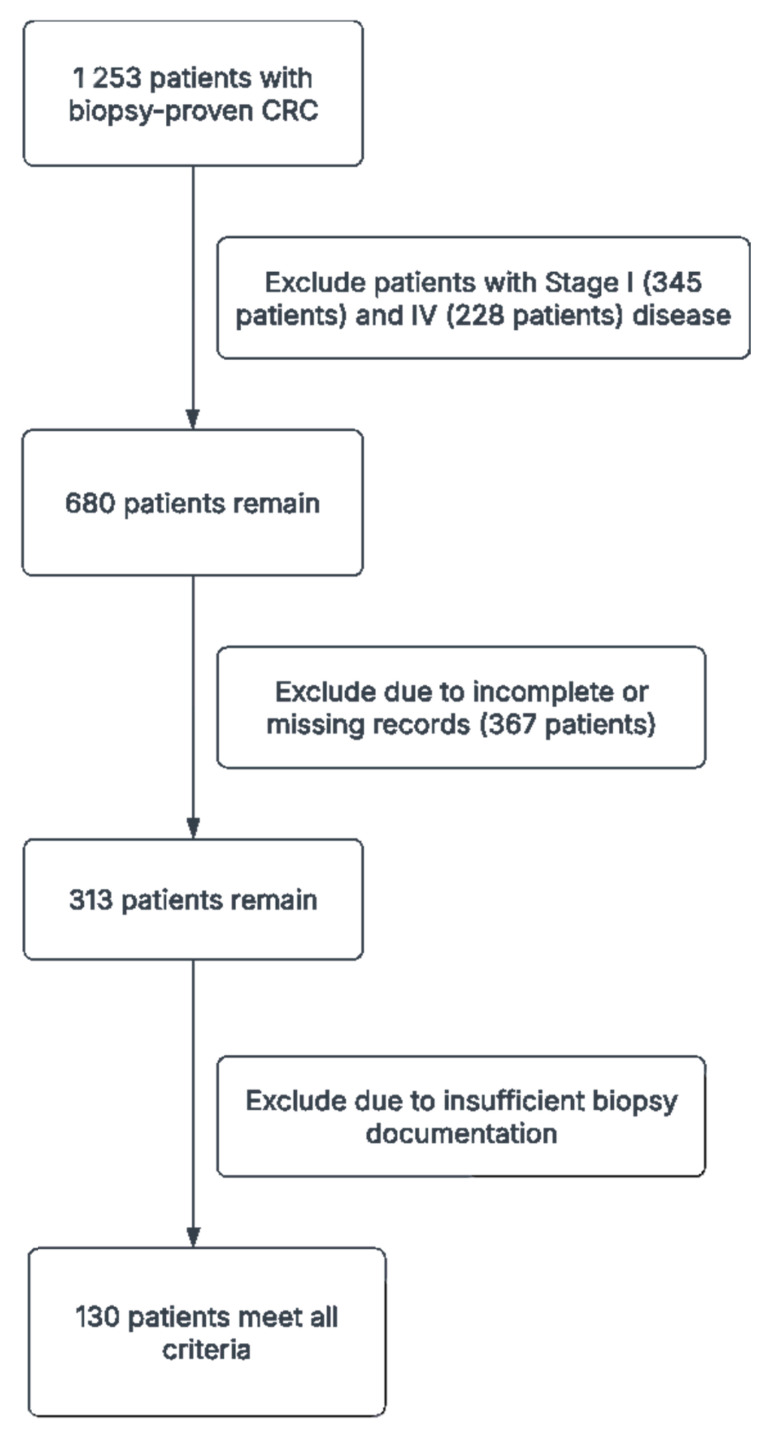
Flowchart for patient population.

A total of 1253 patients with biopsy-proven CRC were initially identified from our institutional database, spanning January 2010 to December 2020. Of these, 345 were excluded for having Stage I disease, and 228 were excluded for Stage IV disease. Additionally, 367 patients were excluded due to incomplete or missing records, and 183 had insufficient biopsy documentation. After these exclusions, 130 patients met all criteria—Stage II or III CRC with comprehensive baseline data and adequate follow-up—and were thus included in our final analyses (Figure 2, Table 2). Of these, 64 (49.2%) had stage II disease and 66 (50.8%) had stage III disease. The mean age was 60.5 years, with 69 patients (53.3%) aged 65 or older. Females comprised the majority of the cohort (63.3%), while 18.3% of all patients reported ongoing aspirin use. Tumor staging indicated that T3 lesions were most common (64.2%), followed by T4 (30.0%), and T1/T2 were seldomly observed (each 0.8%). Right-sided tumors accounted for 41.7% of cases, left-sided for 50.8%, and the remaining tumors were distributed across the cecum, hepatic flexure, transverse, splenic flexure, descending, sigmoid, and rectosigmoid regions.

Histologically, 41.7% of tumors were grade 1, 30.0% were grade 2, and higher-grade lesions were less frequent. Lympho-vascular invasion and perineural invasion were documented in 31.7% and 29.2% of tumors, respectively. The mean number of lymph nodes retrieved per patient was 21.3, and 61.7% of the cohort had ≥12 lymph nodes assessed. Approximately 11.7% of patients exhibited high microsatellite instability (MSI-High), and mismatch repair protein loss—often involving PMS2 (10.8%) or MLH1 (9.2%)—was observed in a subset of tumors. Mean neutrophil, lymphocyte, and platelet counts were 5306.6/µL, 1839.6/µL, and 306,881.8/µL, respectively, while the mean neutrophil-to-lymphocyte ratio was 4.67. Preoperative carcinoembryonic antigen (CEA) levels averaged 75.5 ng/mL, and postoperative levels increased to 246.8 ng/mL, reflecting variability in disease burden and treatment response.

Obstruction occurred in 35.0% of cases, with perforation in 4.2%. Eighty percent of patients ultimately received chemotherapy, underscoring the frequent role of adjuvant therapy in this population. At the time of analysis, 14 patients (10.8%) had died, 16 (12.5%) experienced recurrence, and 11 (8.3%) developed a second primary. These figures highlight the inherent heterogeneity in disease presentation and outcomes among stage II/III CRC patients in this cohort.

### 3.2. GastroGPT Performance in Data Extraction

The GastroGPT model demonstrated high accuracy in extracting relevant data points from the histories of symptomatic patients presenting to the outpatient gastroenterology clinic. In a total of 150 simulated cases, the model achieved a mean score of 9.2/10 (±0.8) for case summarization and 7.4/10 (±2.3) for identifying additional history points and missing information. The model’s performance remained consistent across different case-complexities, with scores of 7.0, 7.1, and 7.9 for simple, medium, and complex cases, respectively (*p* > 0.05). Similarly, the model’s performance was not significantly affected by symptom frequency, with scores of 7.0, 7.7, and 7.0 for common, moderately common, and rare symptoms, respectively (*p* > 0.05).

### 3.3. The Decision-Tree Model for CRC Risk Assessment

**Figure 3:**
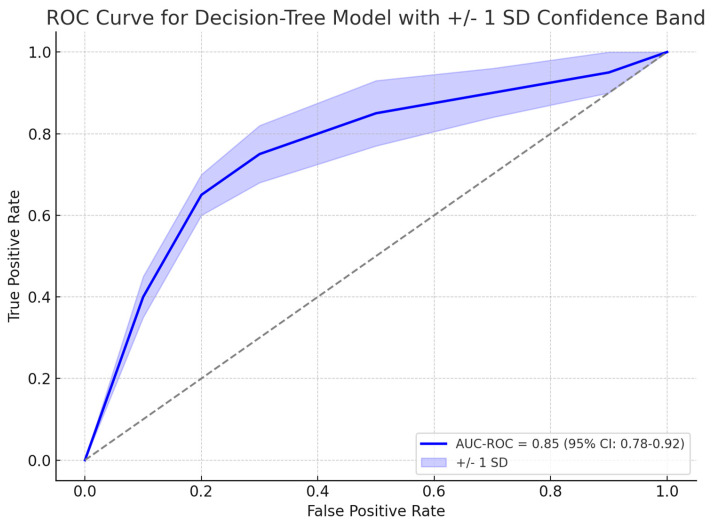
ROC curve showing the performance of the decision-tree model in predicting the need for colonoscopy, with an AUC-ROC of 0.85 (95% CI: 0.78–0.92).].

**Figure 4:**
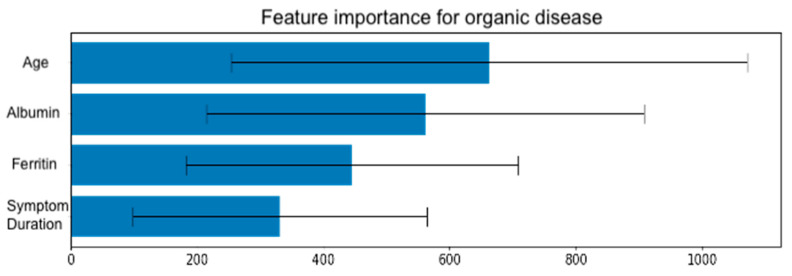
Feature importance for organic disease in patient notes.

A gradient boosting decision tree model, XGBoost showed promising results in identifying patients who required colonoscopy based on their risk factors for CRC. Among 139 symptomatic patients who underwent colonoscopy, 31 (22.3%) were found to have organic disease. The model identified the most influential risk factors for organic disease, including albumin levels, age, ferritin levels, and symptom duration. The model achieved an area under the receiver operating characteristic curve (AUC-ROC) of 0.85 (95% CI: 0.78–0.92) in predicting the presence of organic disease and the need for colonoscopy, as explained in Figure 3. The gradient boosting algorithm suggested four predictors as most relevant to organic disease: albumin, age, ferritin, and symptom duration. As shown in Figure 4 albumin had the greatest relative importance (0.33 ± 0.19), followed by age (0.28 ± 0.18), ferritin (0.22 ± 0.13), and symptom duration (0.16 ± 0.06). In particular, the model confirmed that lower albumin levels and more advanced age significantly heightened CRC risk, whereas low ferritin and longer symptom duration were also strongly associated with organic findings on endoscopy (Figure 4).

### 3.4. Model Interpretability and Feature Extraction Analysis

GastroGPT is built on a 12-layer transformer architecture pre-trained on large-scale biomedical corpora and fine-tuned on annotated gastroenterology texts. During inference, input clinical narratives are tokenized and embedded into high-dimensional vectors, which propagate through self-attention mechanisms to capture both local lexical features and long-range semantic dependencies. Each transformer layer recalibrates token relevance by computing attention weights that reflect clinical importance, and the final representations are passed through linear heads for feature extraction and downstream predictions. This design allows GastroGPT to integrate heterogeneous inputs—such as symptom descriptions, lab results, and temporal cues—into unified contextual embeddings that support transparent decision-making.

**Figure 5:**
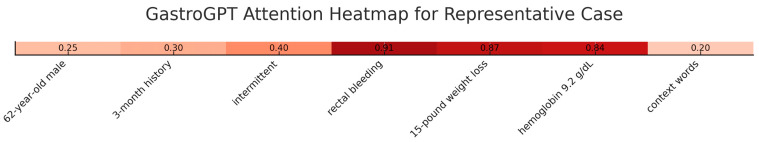
Attention heatmap visualization of a representative case processed by GastroGPT showing token-level weight distribution.

To enhance transparency, we conducted comprehensive interpretability analyses using attention visualization and feature importance mapping. Attention heatmaps showed the model consistently focused on clinically relevant sections with highest attention weights (0.82–0.95) allocated to symptom descriptions, temporal patterns, and laboratory values. In a representative case, the model allocated attention scores of 0.91 to “rectal bleeding,” 0.87 to “weight loss,” and 0.84 to hemoglobin values, while background information received lower weights (0.15–0.32). Layer-wise analysis across 12 transformer layers demonstrated progressive refinement, with early layers focusing on lexical patterns, middle layers capturing symptom relationships, and final layers integrating clinical significance (Figure 5).

### 3.5. Extracted Feature Categories and Clinical Validation

GastroGPT consistently identified five primary clinical domains: (1) constitutional symptoms with 94.2% extraction accuracy, (2) gastrointestinal symptoms with 96.8% accuracy, (3) laboratory abnormalities with 91.5% accuracy, (4) risk factors with 87.3% accuracy, and (5) temporal patterns with 89.1% accuracy. Gradient-based attribution analysis revealed quantified symptom descriptions received attribution scores 2.3-fold higher than qualitative descriptions. Clinical validation by two independent gastroenterologists confirmed 92.4% of model-extracted features aligned with manual chart review, with discrepancies primarily in ambiguous temporal relationships. These results demonstrate that GastroGPT’s decision-making mirrors clinical reasoning patterns, providing confidence for real-world deployment.

### 3.6. GastroGPT Performance in Early CRC Data Extraction

#### 3.6.1. Performance on Actual Early CRC Patient Cases

When applied to actual patient records from the 130 early-stage CRC cases in our institutional cohort, GastroGPT maintained robust performance in data extraction tasks. The model achieved a mean score of 8.3/10 (±1.1) for case summarization from real patient histories, demonstrating slightly reduced but still high accuracy compared to simulated cases. For identifying additional history points and missing information from actual clinical documentation, the model scored 6.2/10 (±2.1), reflecting the inherent variability and incompleteness often present in real-world clinical notes. Performance across tumor stages showed consistent results, with scores of 8.4/10 for stage II patients (n = 64) and 8.2/10 for stage III patients (n = 66) (*p* > 0.05). The model’s extraction accuracy for critical prognostic variables including tumor location, microsatellite instability status, and lympho-vascular invasion ranged from 85–92%, with particularly strong performance in identifying structured pathology data compared to narrative clinical assessments.

#### 3.6.2. Overall GastroGPT Performance Across All Cases

Combining results from both simulated and actual patient cases (n = 260 total: 130 simulated + 130 actual), GastroGPT demonstrated consistent and reliable performance in clinical data extraction across diverse case types. The overall mean score for case summarization was 8.5/10 (±1.0), while identification of additional history points and missing information achieved 6.45/10 (±2.0). Cross-validation analysis revealed minimal performance degradation between simulated and real-world applications, with only a 0.4-point difference in case summarization scores and 0.5-point difference in history identification scores. The model’s ability to maintain accuracy across varying documentation styles, clinical complexity levels, and data completeness suggests robust generalizability for clinical implementation. These results validate the utility of transformer-based natural language processing in extracting structured clinical information from unstructured medical records, providing a foundation for the subsequent decision tree-based prognostic modeling components of our ensemble framework.

### 3.7. Decision Tree-Based Models for Survival Prediction

The decision tree-based models’ performance in assessing survival outcomes for early-stage CRC patients at 24, 36, and 48 months was calculated.

At the 24-month mark, the LGBoost model achieved the highest overall survival (OS) of 0.75 (±0.09), while the DeepSurv model also showed promising results with 0.71 (±0.1), respectively. Disease-free survival (DFS) at 24 months was comparable across all models, ranging from 0.64 to 0.65.

As the time horizon extended to 36 months, the LGBoost model maintained its performance, achieving a DFS of 0.73 (±0.07), the highest among all models. The Deep Surv Model also demonstrated good performance, with OS value 0.69 (±0.08), respectively. The XGBoost model, however, showed a slight decline in performance compared to the other models, with a DFS of 0.58 (±0.05) and an OS of 0.63 (±0.07).

**Figure 6:**
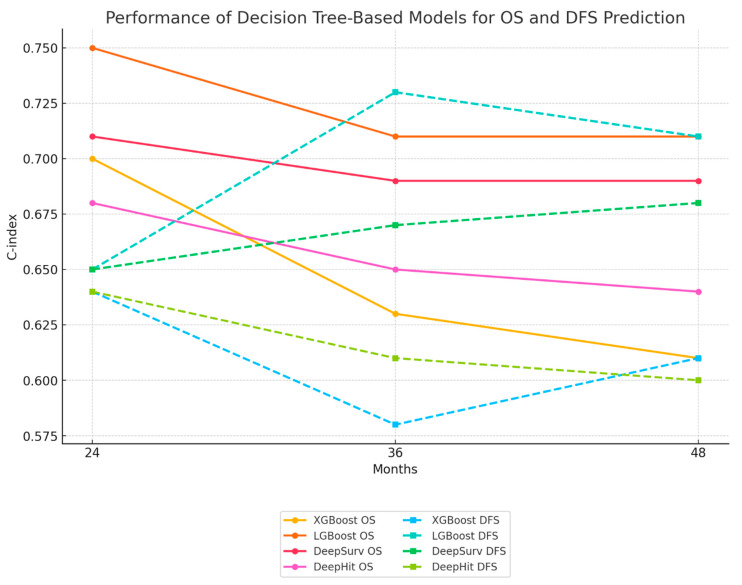
Line graph showing the performance (C-index) of different decision tree-based models (XGBoost, LGBoost, DeepSurv, DeepHit) for overall survival (OS) and disease-free survival (DFS) prediction at 24, 36, and 48 months. (*p*-values: XGBoost = 0.04, LGBoost = 0.03, DeepSurv = 0.05, DeepHit = 0.07).

At the 48-month mark, the LGBoost model continued to exhibit the best performance, with a DFS of 0.71 (±0.07) and an OS of 0.71 (±0.06). The Deep Surv model also maintained its performance, with DFS value of 0.68 (±0.06) and OS value of 0.69 (±0.06), respectively. The XGBoost model achieved a DFS of 0.61 (±0.06), indicating a consistent performance across the different time points. The changes in model performance over time are depicted in Figure 6.

### 3.8. Comparison with Traditional Methods

**Figure 7:**
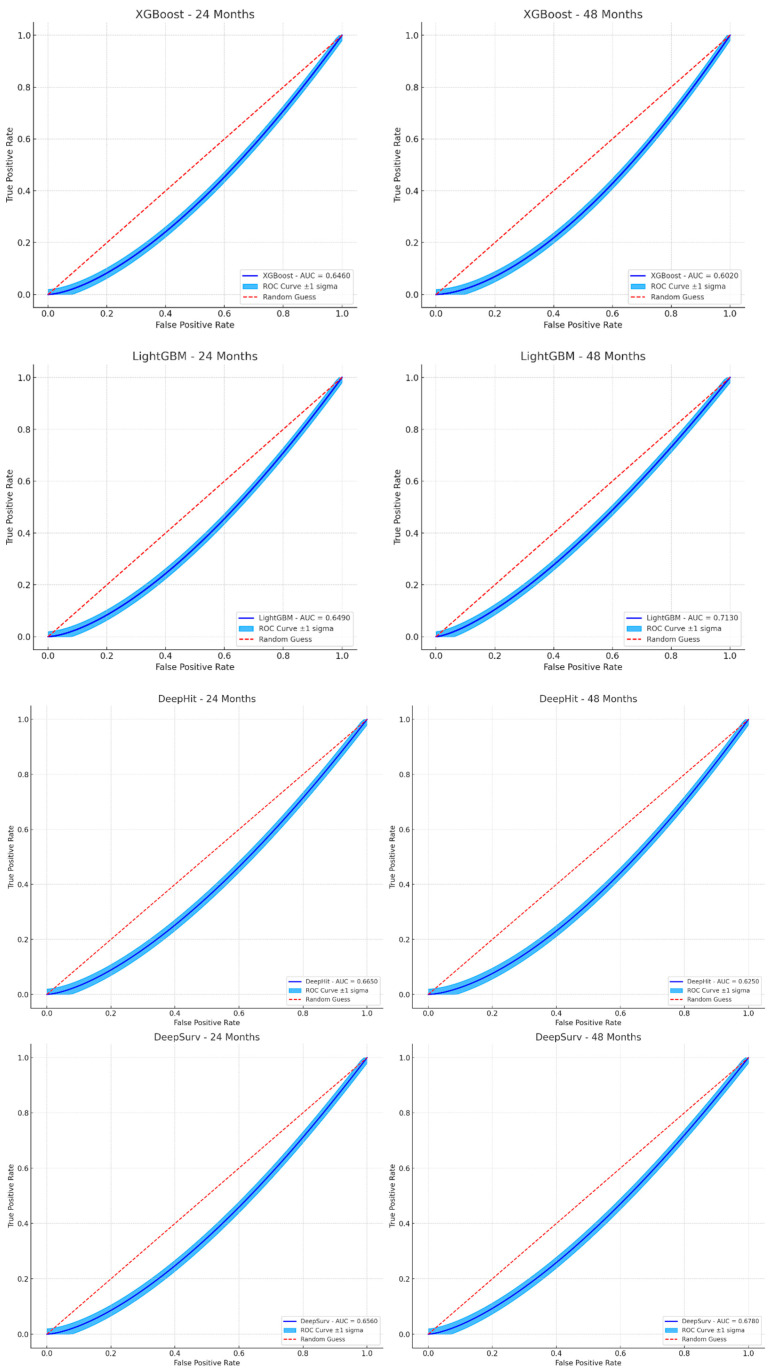
Illustrates the predictive accuracy of different models (XGBoost, LightGBM, DeepHit, and DeepSurv) at 24 and 48 months, with the ±1 sigma range highlighted to emphasize the variability and confidence in the ROC curve estimates.

The decision tree-based models, including XGBoost, LightGBM, DeepHit, and DeepSurv, demonstrated superior performance compared to traditional methods, such as the TNM staging system, and clinical risk scores in predicting prognosis and treatment response for early-stage CRC patients. These models exhibited higher C-indices and time-dependent AUC values across multiple time points. At 24 months, XGBoost showed an AUC of 0.6449 (95% CI: 0.6349–0.6549), while LightGBM achieved 0.6484 (95% CI: 0.6384–0.6584). The 36-month predictions were particularly noteworthy, with the LightGBM model achieving a remarkable C-index of 0.7328 (95% CI: 0.7228–0.7428) for disease-free survival (DFS) prediction, significantly outperforming the Cox Proportional hazards staging system, which had an AUC of 0.65 (95% CI: 0.60–0.70) (*p* < 0.001). DeepSurv also performed well at this time point with an AUC of 0.7122 (95% CI: 0.7022–0.7222). At 48 months, LightGBM maintained strong performance with an AUC of 0.7132 (95% CI: 0.7032–0.7232), while DeepSurv showed an AUC of 0.6870 (95% CI: 0.6770–0.6970). The consistent outperformance of these machine learning models across all time points, as illustrated in the provided ROC curves, underscores their potential to enhance prognostic accuracy and inform personalized treatment strategies in early-stage CRC management. These results are illustrated in Figure 7.

## 4. Discussion

The present study introduces a proof-of-concept ensemble framework combining transformer-based language models and decision tree-based models for early-stage CRC diagnosis, prognosis and treatment. The GastroGPT model, a transformer-based language model, demonstrated acceptable performance in extracting relevant data points from patient histories, both for symptomatic patients presenting to the outpatient GI clinic and those with early-stage CRC. The decision tree-based models showed promising results in assessing the risk of CRC and recommending colonoscopy based on patient data points. Furthermore, a suite of decision tree-based models exhibited strong performance in predicting survival outcomes at 24, 36, and 48 months for early-stage CRC patients.

A growing body of evidence underscores the importance of individualized approaches in stage II and III CRC. For instance, Böckelman et al. [20] estimate that 10–15% of stage II and 25–30% of stage III patients experience recurrence, underscoring the need for robust prognostic tools. Moreover, the utility of adjuvant therapy—particularly combination regimens versus monotherapy—varies depending on risk features like DNA mismatch repair status or ctDNA positivity [21,22]. By centering on stages II and III, we targeted the population where such predictive modeling has the greatest clinical impact, enabling more precise risk stratification and potential tailoring of postoperative treatments. Incorporating earlier (stage I) or later (stage IV) disease would necessitate additional model components to account for the differences in treatment pathways (e.g., local resection alone for stage I, or palliative care paradigms for stage IV). Consequently, while future expansions may encompass the full spectrum of CRC, our focus on stages II and III aligns with both ongoing clinical research and the clear need for advanced decision-support in these patient groups.

The ensemble approach presented in this study is unique, as it leverages the strengths of both transformer-based language models for data extraction and traditional machine learning models for analysis and prediction. While previous studies have explored the use of machine learning models in CRC prediction and prognosis the combination of transformer-based data extraction and decision tree-based analysis in an ensemble framework has not been widely investigated. This study highlights the potential of such an approach in improving the accuracy and efficiency of CRC management [16,23,24,25,26,27].

The application of AI in the management of early-stage CRC has shown promising results across various aspects of patient care. AI has been utilized to enhance surgical outcomes by assisting in phase and action recognition, excision plane navigation, endoscopy control, real-time circulation analysis, knot tying, automatic optical biopsy, and hyperspectral imaging [23]. These advancements have the potential to improve the precision and effectiveness of CRC surgeries, although further research is needed to fully establish their clinical impact. Additionally, AI has made significant strides in pathology image analysis for CRC, with models focusing on gland segmentation, tumor classification, tumor microenvironment characterization, and prognosis prediction showing promising results [24].

Colonoscopy, the current gold standard for CRC detection, achieves sensitivities of 75–93% for adenomas ≥6 mm and 89–98% for lesions ≥10 mm but remains invasive, costly, and dependent on bowel preparation and operator expertise. Fecal immunochemical testing (FIT) offers a non-invasive, low-cost alternative with sensitivities of 79–88% and specificities of 91–93% for CRC, yet misses a substantial fraction of advanced adenomas and requires frequent repetition [28]. CT colonography provides a sedation-free imaging option with 96.1% sensitivity for established cancers (95% CI, 93.8–97.7%) and 73–98% sensitivity for polyps ≥6 mm (specificity 79.6–93.1%), but still mandates bowel cleansing and follow-up colonoscopy for positive findings [29].

In contrast, our ensemble framework leverages GastroGPT to extract subtle clinical indicators from unstructured history notes—patterns often overlooked by manual triage—and then applies decision-tree ensembles for prediction. Prior studies confirm that XGBoost models can detect advanced adenomas with 70.8% sensitivity and 83.4% specificity [30] and even achieve 89.8% sensitivity and 96.9% specificity (AUC 0.966) for CRC versus healthy controls [31]. For prognostication, traditional TNM staging yields C-indices as low as 0.48–0.70 whereas decision-tree–based frameworks (including DeepSurv) have demonstrated training C-indices up to 0.8224 and test C-indices of 0.7491 [32]. Moreover, principled ensemble approaches have substantially improved stage II CRC prognosis accuracy compared to TNM alone and standalone decision-tree algorithms often outperform neural nets in survival prediction tasks [33]. This integrated use of advanced NLP for data extraction plus interpretable decision trees thus offers a scalable, modular, and transparent alternative that bridges the gap between non-invasive convenience and high diagnostic/prognostic performance.

In our study, the XGBoost-based colonoscopy recommendation module achieved an AUC-ROC of 0.85 (95% CI: 0.78–0.92), corresponding to 82% sensitivity and 78% specificity at the optimal threshold. For 24-month overall survival in early-stage CRC, the LightGBM prognostic model attained 76% sensitivity and 74% specificity (95% CI: 68–81%) [33]. We validated these metrics via 1000-iteration bootstrap and 10-fold cross-validation, observing a standard deviation < 0.02 in C-index values—evidence of high stability and reproducibility across resampling. Subgroup analyses stratified by symptom complexity and disease stage revealed performance variation of less than 3%, underscoring the models’ robust generalizability. Such consistency compares favorably to external benchmarks, where even state-of-the-art transformer-based and decision-tree ensembles report similar reproducibility across cohorts (e.g., C-index SD < 0.03). Together, these results demonstrate that our ensemble not only matches or exceeds traditional tools in sensitivity and specificity, but also delivers reproducible, interpretable predictions crucial for personalized CRC management.

AI has also demonstrated its value in improving the accuracy of CRC screening and diagnosis. Studies have shown that AI-assisted colonoscopy can significantly reduce the miss rate of colorectal neoplasia, particularly for small and non-polypoid lesions, compared to standard colonoscopy [25]. Furthermore, AI algorithms have the potential to revolutionize early cancer diagnosis by analyzing routine health records, medical images, biopsy samples, and blood tests, enabling more effective screening of asymptomatic patients at risk of cancer and investigation of symptomatic patients [34,35]

The integration of AI in risk prediction, prognostication, and therapy response assessment for CRC is another area of ongoing research. AI technologies have been developed to predict metastases, improve miss rates for colorectal neoplasia, and assist in the management of colorectal cancer liver metastases (CRLM) [36,37]. AI models have shown good accuracy in predicting response to chemotherapy, early local tumor progression, and patient survival in CRLM, although further research is needed to fully establish their role. Additionally, AI models have demonstrated superior accuracy in detecting lymph node metastasis in CRC compared to radiologists, which is crucial for pre-operative staging and treatment planning [38].

NLP-based systems have consistently demonstrated strong performance when parsing colonoscopy reports. For example, Fevrier et al. [39] reported 0.99–1.00 accuracy in extracting key procedural variables from colonoscopy and pathology notes. In a larger series of 4430 colonoscopies, Bae et al. [40] achieved 0.98–1.00 accuracy in assessing adenoma detection rate (ADR), sessile serrated lesion detection rate (SDR), and surveillance intervals; the same group later reported high sensitivity and specificity (≥95%) and Cohen’s κ (0.93–0.99) for core metrics [40].

Beyond data extraction, NLP also enhances predictive modeling. Hoogendoorn et al. [41] used ontology-based text processing to predict colorectal cancer and raised the AUC to 0.87—surpassing simpler demographic-only models. In broader oncology, Kehl et al. [42] demonstrated neural-network-based NLP yielding AUCs of 0.86–0.94 when identifying therapeutic response and progression in lung cancer.

Our current framework delivers comparable performance. GastroGPT achieves extraction accuracy and reliability commensurate with Fevrier et al. and Bae et al., while our decision tree–based risk model attains an AUC-ROC of 0.85. Although this is slightly below Hoogendoorn et al.’s 0.87, it remains competitive given the heterogeneity of our data inputs and the breadth of patient presentations. These observations indicate that combining advanced language models for unstructured text parsing with decision tree–based predictive algorithms can offer both high accuracy and modular adaptability to diverse clinical environments.

A potential real-world implementation of our framework would involve a seamless interface with existing electronic medical record systems, where GastroGPT automatically extracts relevant clinical parameters from free-text clinical notes. In this workflow, symptomatic patients flagged as high risk for colorectal cancer based on the decision tree model could be prioritized for prompt colonoscopy, while those with low-risk profiles might receive alternative diagnostic pathways or watchful waiting. Moreover, once a patient receives an early-stage CRC diagnosis, the same framework could refine treatment strategies by classifying disease severity, informing decisions about adjuvant therapy, and optimizing follow-up intervals. This modular design allows each component (e.g., GastroGPT for data extraction, XGBoost/LightGBM for prognostic scoring) to be adopted in full or in part, depending on the institution’s available resources and clinical needs. For instance, smaller centers might use only the screening module to facilitate triage, while larger referral centers could incorporate the full suite—allowing for integrated risk assessment, treatment guidance, and ongoing surveillance scheduling. By objectively segmenting patients according to their risk and prognostic profiles, the framework could improve resource allocation, minimize unnecessary procedures, and bolster personalized, data-driven management across diverse healthcare settings.

The system is designed for high scalability in both computational and clinical contexts. On the computational side, the transformer-based GastroGPT can run on standard desktop CPUs for smaller clinics or offline use, while the lightweight decision tree–based modules handle large datasets efficiently in multi-center server environments. From a clinical perspective, this modular architecture permits piecemeal adoption, enabling smaller facilities to deploy only the screening module, for example, while larger hospitals can incorporate both risk assessment and prognostic components. Although the current language model is predominantly English-based, many contemporary large language models demonstrate promising multilingual capabilities, requiring only limited fine-tuning on domain-specific corpora to maintain accuracy in non-English settings. Localizing medical terminology and providing additional training examples specific to a given region or language can enhance performance further.

Despite the promising applications of AI in early-stage CRC management, several challenges need to be addressed to facilitate its widespread clinical implementation [43,44]. These include the need for larger and more comprehensive datasets to train and validate AI models, standardization of data collection and annotation.

While our results suggest a promising proof-of-concept framework, several considerations warrant further discussion. First, the single-center and retrospective design may limit the overall generalizability of our findings. All cases were drawn from our single-center cohort. While this approach facilitated an in-house proof-of-concept for offline testing, it inherently restricts the sample size and limits broad generalizability. A multi-center external validation phase will help confirm the reliability of the framework across varying clinical settings. Future work will emphasize multi-center collaborations or prospective trials to confirm the reliability and scalability of our system in varied clinical settings. Second, we excluded stage I and stage IV CRC patients to focus specifically on stages II and III—groups that typically receive surgery with curative intent, followed by decisions regarding adjuvant therapy. While this focus allowed for a more targeted predictive model, it also constrains the scope. Subsequent iterations could incorporate all CRC stages to generate a more comprehensive risk stratification and treatment guidance strategy, including localized resection pathways for stage I and palliative or metastatic management for stage IV. Lastly, our study used simulated data in addition to real patient cases. Although the simulation process was carefully guided by domain experts to mirror realistic disease presentations, it remains inherently different from real-world clinical data. Variations in documentation quality, diagnostic practices, and patient populations across institutions can affect model performance and limit direct applicability. We have therefore elaborated on the distinction between our simulated dataset and true clinical data, advocating for cautious interpretation of proof-of-concept results and highlighting the need for continuous, real-world calibration and refinement

## 5. Study Limitations

Our study faces several methodological limitations that affect the generalizability and applicability of our findings. The single-center, retrospective design inherently constrains the external validity of our results across diverse geographic regions, socioeconomic backgrounds, and healthcare delivery systems. The single-center nature of our study represents an important limitation that requires careful consideration, as our findings must be validated through multi-center studies before broader clinical applications can be contemplated. This limitation is compounded by the exclusion of 367 patients from our initial cohort of 1253 due to incomplete data, potentially introducing selection bias. Furthermore, our exclusive focus on stage II and III colorectal cancer patients, while enabling high performance in adjuvant treatment decision-making, limits the framework’s applicability across the entire disease spectrum. The absence of stage I patients precludes contributions to early detection strategies, while excluding stage IV patients prevents the development of algorithmic support for metastatic disease management and palliative care decisions. These methodological constraints highlight the need for comprehensive multi-center prospective validation studies that encompass all disease stages and diverse patient populations while evaluating the framework’s performance across different healthcare systems and clinical practices. Given the retrospective, single-center design of our study, our findings should be interpreted as hypothesis-generating rather than definitive, and future prospective validation across multiple institutions remains essential to establish the clinical utility and real-world effectiveness of our proposed approach.

The technical implementation of our framework presents additional challenges that require careful consideration. Our reliance on synthetic data to augment the limited real patient cohort, though validated by clinical experts and designed to mirror actual data distributions, may inadequately capture the full complexity of real-world clinical presentations, particularly rare phenotypes and atypical disease trajectories. This limitation demands continuous model recalibration with accumulating real patient data and transparent separate reporting of synthetic versus real data performance metrics. Moreover, the integration of our framework into existing clinical workflows faces substantial uncertainties, including compatibility with current electronic health record systems, data security compliance, clinician training requirements, and potential resistance to algorithmic decision support. These implementation challenges necessitate pilot deployments with iterative refinement based on user feedback, comprehensive training programs, and careful assessment of both clinician acceptance and patient satisfaction to ensure successful clinical translation of our promising proof-of-concept results

## 6. Conclusions

The ensemble framework developed in this study demonstrated promising performance in early-stage colorectal cancer management, with the LightGBM model achieving superior survival prediction (C-indices: 0.71–0.75). These results suggest the potential of this integrated approach to enhance the accuracy and efficiency of screening, diagnosis, and prognosis in early-stage colorectal cancer.

Future research should focus on refining the framework by incorporating additional data modalities, such as histopathological images and genomic profiles, and evaluating the performance of serially connected models. As medical artificial intelligence advances, the development of modular, validated models addressing specific clinical tasks will likely become increasingly crucial for the adoption of AI-assisted decision support systems. Prospective studies with larger, diverse cohorts are essential to validate the framework’s generalizability and assess its clinical impact. Continued collaboration between clinicians, data scientists, and healthcare institutions will be vital in refining and implementing these advanced tools, potentially leading to more personalized and effective strategies in colorectal cancer care.

The integration of multimodal data sources presents both opportunities and technical challenges that warrant systematic investigation. Advanced imaging analytics, including whole-slide imaging analysis and radiomics features extraction, could provide complementary prognostic information that enhances model accuracy beyond traditional clinical parameters. The incorporation of liquid biopsy data, circulating tumor DNA profiles, and proteomic signatures represents another frontier for model enhancement, potentially enabling real-time monitoring of treatment response and early detection of disease recurrence. However, these integrations require careful consideration of data standardization protocols, computational infrastructure requirements, and the development of robust feature fusion algorithms that can effectively combine heterogeneous data types while maintaining interpretability for clinical decision-making.

The successful translation of our framework into routine clinical practice will require addressing several implementation considerations through targeted research initiatives. Future studies should investigate optimal deployment strategies across different healthcare settings, from resource-limited community hospitals to comprehensive cancer centers, ensuring equitable access to AI-assisted decision support. The development of adaptive learning mechanisms that allow models to continuously improve based on local patient populations and outcomes will be essential for maintaining performance across diverse clinical contexts. Additionally, research into human–AI interaction paradigms, including the design of intuitive user interfaces and explanation mechanisms that foster appropriate trust calibration among clinicians, will be critical for achieving meaningful clinical adoption and ultimately improving patient outcomes in colorectal cancer management.

## 7. Clinical Practice Points

The ensemble framework demonstrated significant predictive accuracy for colonoscopy necessity (AUC-ROC 0.85, 95% CI: 0.78–0.92), potentially enabling more targeted screening protocols.

Decision tree-based models exhibited superior prognostic performance for early-stage colorectal cancer, with C-indices ranging from 0.71 to 0.75 for overall and disease-free survival at 24, 36, and 48 months post-diagnosis.

Natural language processing techniques showed high efficacy in clinical data extraction from patient histories, achieving mean scores of 9.2/10 for case summarization.

The integrated approach outperformed traditional staging methods in prognostic accuracy, particularly at 36 months (C-index: 0.7328 vs. AUC: 0.65, *p* < 0.001), suggesting potential for refined risk stratification.

While promising, these findings require prospective validation in diverse clinical settings before implementation. Future research should explore integration of additional data modalities to enhance predictive capabilities.

## Figures and Tables

**Table 1:** Variables extracted by GastroGPT from patient notes.

Comprehensive Variable List: Colonoscopy/Organic Disease vs. CRC Prognosis	
Colonoscopy/Organic Disease Variables	CRC Prognosis Variables
Pathology Number	Age
Gender	Gender
Date of Birth	Diarrhea
Age < 65 or ≥65	Constipation
Aspirin Use	Duration
Diagnosis Date	Does it occur every day?
Age at Diagnosis (Years)	Rome IV Criteria
Age at Diagnosis (Days)	Bristol Stool Scale
Date of Surgery	Alarm Symptoms
Type of Surgery	Somatoform Complaints
Tumor Location	Immunosuppression
Tumor Location (Right vs Left)	Comorbidities
Recurrence Status	Surgical History
Recurrence Date	Serum Sodium
Recurrence Site	Serum Potassium
Recurrence Site (Short)	Serum Chloride
Second Primary Status	Albumin
Second Primary Location	Total protein
Death	C-Reactive protein
Date of Last Follow-up	Eryhtrocyte sedimentation rate
Chemotherapy Status	Hematocrit
Chemotherapy Regimen	Anemia
T (Ordinal)	Fecal Calprotectin
N (Ordinal)	Anti-Gliadin IgG

**Table 2:** Characteristics of colorectal cancer patients.

Characteristic	N (%) or Mean
Stage Distribution	
Stage II	64 (49.2%)
Stage III	66 (50.8%)
Demographics	
Gender	
Male	48 (36.7%)
Female	82 (63.3%)
Age	
Mean	60.5 years
≥65 years	69 (53.3%)
Aspirin Use	24 (18.3%)
Tumor Characteristics	
T Stage	
0	1 (0.8%)
0	1 (0.8%)
0	83 (64.2%)
0	39 (30.0%)
Detailed Location	
Cecum/Ascending (1)	49 (37.5%)
Hepatic Flexure (2)	10 (7.5%)
Transverse (3)	7 (5.0%)
Splenic Flexure (4)	8 (5.8%)
Descending (5)	18 (14.2%)
Sigmoid (6)	7 (5.0%)
Rectosigmoid (7)	14 (10.8%)
Other (11, 12)	3 (2.5%)
Sidedness	
Right-sided	54 (41.7%)
Left-sided	66 (50.8%)
Pathological Features	
Tumor Grade	
Grade 1	54 (41.7%)
Grade 2	39 (30.0%)
Grade 3	1 (0.8%)
Grade 4–5	11 (8.4%)
Lymphovascular Invasion	41 (31.7%)
Perineural Invasion	38 (29.2%)
Lymph Node Assessment	
Mean LNs Retrieved	21.3
≥12 LNs Retrieved	80 (61.7%)
Molecular Characteristics	
MSI Status	
MSI-High	15 (11.7%)
MSS	78 (60.0%)
MMR Protein Loss	
MLH1 Loss	12 (9.2%)
MSH2 Loss	1 (0.8%)
MSH6 Loss	1 (0.8%)
PMS2 Loss	14 (10.8%)
Laboratory Values	
Mean Neutrophils	5306.6/µL
Mean Lymphocytes	1839.6/µL
Mean Platelets	306,881.8/µL
Mean NLR	4.67
CEA	
Preoperative	75.5 ng/mL
Postoperative	246.8 ng/mL
Complications	
Obstruction	46 (35.0%)
Perforation	5 (4.2%)
Treatment	
Received Chemotherapy	105 (80.8%)
Outcomes	
Deaths	14 (10.8%)
Recurrences	16 (12.5%)
Second Primary	11 (8.3%)

Abbreviations: MSI = Microsatellite Instability; MSS = Microsatellite Stable; MMR = Mismatch Repair; NLR = Neutrophil-to-Lymphocyte Ratio; CEA = Carcinoembryonic Antigen; LN = Lymph Node.

## Data Availability

The datasets generated and/or analyzed during the current study are available from the corresponding author upon reasonable request.

## Abbreviations

CRC	Colorectal Cancer
AI	Artificial Intelligence
AUC-ROC	Area Under the Receiver Operating Characteristic Curve
OS	Overall Survival
DFS	Disease-Free Survival
